# Spontaneous body contractions are modulated by the microbiome of *Hydra*

**DOI:** 10.1038/s41598-017-16191-x

**Published:** 2017-11-21

**Authors:** Andrea P. Murillo-Rincon, Alexander Klimovich, Eileen Pemöller, Jan Taubenheim, Benedikt Mortzfeld, René Augustin, Thomas C. G. Bosch

**Affiliations:** Zoological Institute and Interdisciplinary Research Centre Kiel Life Science, University of Kiel, 24098 Kiel, Germany

## Abstract

Spontaneous contractile activity, such as gut peristalsis, is ubiquitous in animals and is driven by pacemaker cells. In humans, disruption of the contraction pattern leads to gastrointestinal conditions, which are also associated with gut microbiota dysbiosis. Spontaneous contractile activity is also present in animals lacking gastrointestinal tract. Here we show that spontaneous body contractions in *Hydra* are modulated by symbiotic bacteria. Germ-free animals display strongly reduced and less regular contraction frequencies. These effects are partially restored by reconstituting the natural microbiota. Moreover, soluble molecule(s) produced by symbiotic bacteria may be involved in contraction frequency modulation. As the absence of bacteria does not impair the contractile ability itself, a microbial effect on the pacemakers seems plausible. Our findings indicate that the influence of bacteria on spontaneous contractile activity is present in the early-branching cnidarian hydra as well as in Bilateria, and thus suggest an evolutionary ancient origin of interaction between bacteria and metazoans, opening a window into investigating the roots of human motility disorders.

## Introduction

Spontaneous contractile activity of the gut, known as peristalsis, is ubiquitous in animals with a gastrointestinal tract. This intrinsic activity, which appears essential to animal’s life, is caused by rhythmic electrical pulses generated by pacemaker cells. These cells generate periodic depolarizations without the need of external stimuli and transmit their depolarizing waves to effector cells. In vertebrates, the pacemakers underlying the peristaltic movements of the digestive tract are the interstitial cells of Cajal (ICC)^1^. These cells spread depolarizing currents, known as slow waves, via gap junctions to smooth muscular cells that subsequently contract. Although the generation of the pacemaker activity *per se* does not depend on neuronal input, its modulation, for instance the speed and amplitude of the bursts, is under neuronal control^2^.

The importance of pacemaker systems in mammals has been demonstrated by functional alterations, which change gut motility patterns, leading to pathological conditions and sometimes death. For instance, mutant mice lacking ICC die *in uterus*, while mutants with diminished numbers of ICC fail to produce slow waves, resulting in abnormal intestinal motility^1,3^. In humans, disruption of the ICC network is associated with several gastrointestinal motility disorders, including inflammatory bowel conditions, chronic constipation and intestine pseudo-obstruction^4–6^.

In addition to the fundamental role of pacemakers in gut motility, increasing evidence suggests that the gut microbiota contributes to the regulation of the contractile activity. The relationship between intestinal motility and the gut microbiota has been known for decades^7–9^ and its role affecting animal physiology is increasingly appreciated^10–12^. For instance, in germ-free zebrafish and mice, the gut remains underdeveloped and displays dysmotility^13–16^. In humans, gastrointestinal motility disorders such as inflammatory bowel syndrome and jejunal diverticulosis have been associated with small-intestinal bacterial overgrowth and a severely disturbed gut microbiota^17,18^. However, the true nature of the interaction between the microbiota and motility and the impact of microbes on pacemaker activity in the gut is not known.

In invertebrates, the pacemaker cells regulating rhythmic contractions are neurons embedded in neuronal networks known as central pattern generators (CPG)^19,20^. In insects, CPGs located in different ganglia of the stomatogastric nervous system (SGNS) interact with each other to control motility of the foregut^21,22^. In addition, it has been shown that interrupting the connection among the SGNS ganglia impairs the pacemaker activity, which in turn disrupts the foregut contractions^23,24^. Similar CPG systems are also present in molluscs^25^.

The spontaneous and rhythmic contractile activity which in vertebrates is responsible for gastrointestinal peristalsis, is also observed in animals without a gastrointestinal tract, such as sponges and cnidarians^26,27^. In corals, jellyfish, colonial hydrozoans as *Hydractinia*, and hydra, this motility appears to be involved in multiple functions including morphogenesis, regeneration, osmoregulation, respiration and propulsion^26,28–33^. Therefore, regardless of the species, this spontaneous activity appears essential to the organisms’ homeostasis and vital processes.

**Figure 1:**
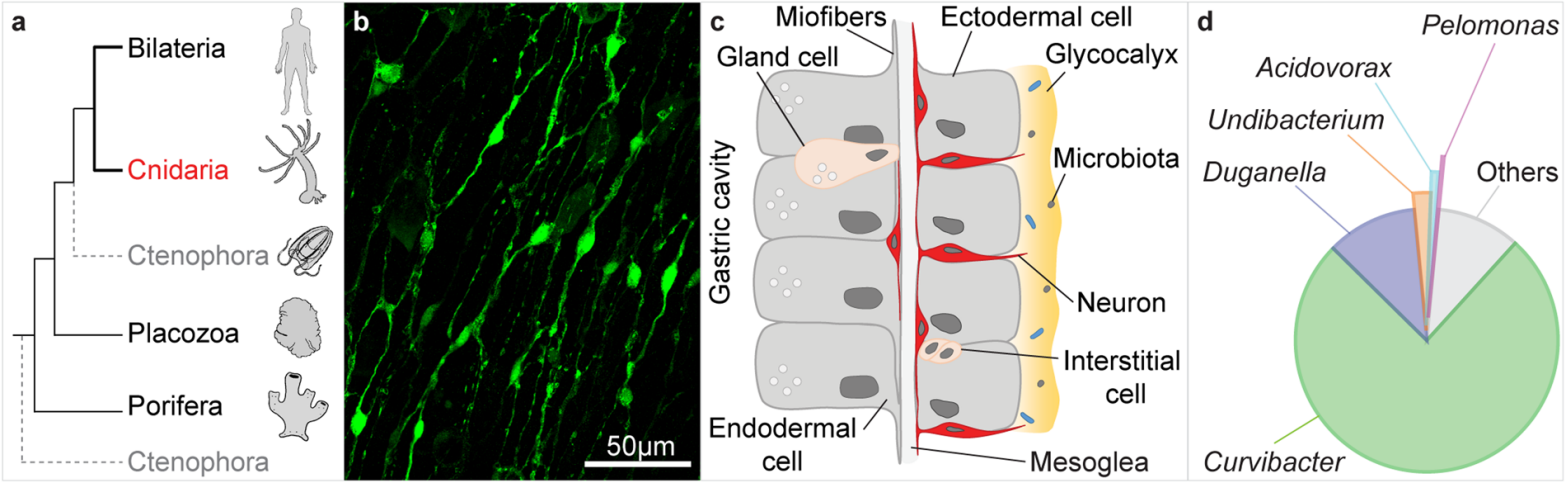
*Hydra* as a model to study host-symbiont interactions. (**a**) *Hydra* belongs to the phylum Cnidaria, the sister group of bilateria. (**b**) *Hydra* has a simple nerve net. Here ganglion neurons in the body column are revealed by expression of RFP (green pseudo-color) under the actin promoter. (**c**) The body wall of *Hydra* is composed of three cell lineages: the ectodermal and the endodermal epithelia that are separated by an extracellular matrix (mesoglea), and the lineage of interstitial cells that differentiate into neurons, gland cells, cnidocites and gametes. The outer surface of the ectoderm is covered by a glycocalyx, the habitat of symbiotic bacteria. The endoderm lining the gastric cavity, is free of glycocalyx and stable microbiota. (**d**) 90% of *H. vulgaris* AEP microbiota is composed by five bacterial strains, with less abundant strains making up the remainder.

The phylum Cnidaria emerged about 700 mya^34,35^ and is one of the first animal groups to possess a nervous system (Fig. 1a,b)^36,37^. Spontaneous contractions in these animals are controlled by central pattern generators too^38–41^. The cnidarian *Hydra* has a diploblastic body column (Fig. 1c) that forms a tube with an apical head and a basal foot. The body column lumen, referred to as the gastric cavity, has digestive functions and can be considered as an evolutionary ancient intestine^42^. Similar to other animals, hydra display spontaneous rhythmic contractile behaviour, suggesting the existence of pacemaker activity. Although the cell population(s) responsible for generating this activity have not been characterized yet, the evidence suggests that they are neurons, as the spontaneous contractions are abolished in nerve-free hydras^43^ and drastically reduced when gap junction communication among neurons is inhibited^44^. Moreover, electrophysiological studies have located spontaneous rhythmical electrical activity in the head and the foot region^45–48^. Environmental factors, such as light and chemical signals (*e.g*. glutathione) affect contraction frequency, with pacemaker systems suggested as direct targets of these inputs^45,49^.

As in other animals, the epithelial surface of hydra is densely colonized by a stable and relatively simple bacterial community (Fig. 1e,d), whose presence is critical for tissue homeostasis and health of the animal^50,51^. Here we demonstrate an additional function of the microbiome of the standard laboratory strain *H. vulgaris AEP*: the symbiotic bacteria influence the spontaneous contractions, likely by modulating the pacemaker activity.

## Results

### Spontaneous contraction behaviour is altered in germ-free animals

**Figure 2:**
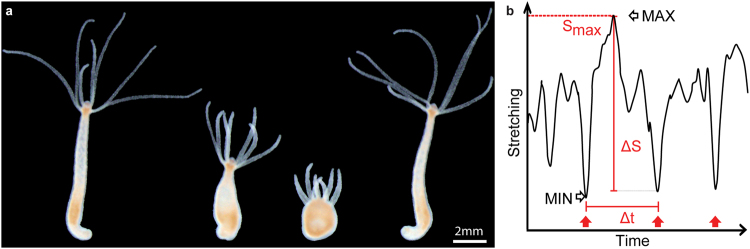
Spontaneous contractile behaviour of *Hydra*. (**a**) Sequence of pictures demonstrating the spontaneous contraction of the body column, hydra’s most common behaviour. (**b**) Spontaneous contractions assessment. The contractile behaviour was described in terms of contraction frequency, defined as the number of full body contractions (MIN) occurred in one hour (red arrows), the time interval between two consecutive contractions Δt, the stretching capacity S_max_, which is the maximum body elongation (MAX) achieved after a contraction and the contractile capacity ΔS, the difference in a polyp’s shape between MAX and MIN states.

In order to study the impact of symbiotic bacteria on the contractile activity of hydra, we recorded time-lapse videos of individual, undisturbed, light-adapted animals, harbouring undisrupted microbiota. These animals are further referred to as control polyps. An example video demonstrating the spontaneous contractile behaviour is available as online supplementary information. Images demonstrating different stages of a full body contraction are shown in Fig. 2a. We assessed the contraction frequency (number of contractions/hour) and time interval between two consecutive contractions (Δt, Fig. 2b). Control polyps contracted on average 7.8 ± 0.1 times per hour (mean ± SE). These contractions appeared in a regular temporal pattern with an average interval of 7.32 ± 0.14 min, and 75% of consecutive contractions occurred within 10 min-interval.

**Figure 3:**
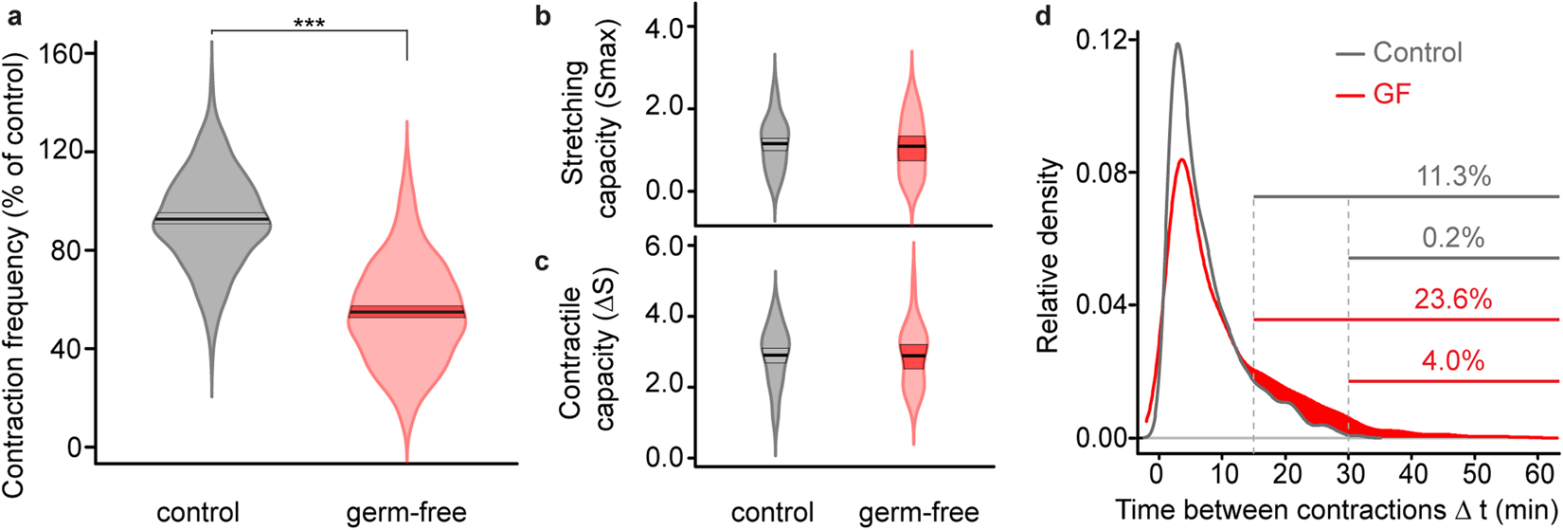
Absence of bacterial microbiota affects *Hydra* spontaneous contraction behaviour. (**a**) Violin plot showing the contraction frequency reduction in GF (*n* = 236) compared to control (*n* = 271) animals (*x* ^2^ = 426, df = 1, ***P ≤ 0.001; linear mixed effects model with replicate as random effect). (**b**,**c**) Epithelium ability to elongate given by stretching capacity, Smax (**b**) and contractile capacity ΔS (**c**) is not impaired in GF animals, evidenced by the lack of statistical difference in Smax and ΔS between GF and control animals (**b**, *t* = 0.07, df = 47.63, *P* = 0.94; maximum elongated states in GF *n* = 22, 0.96 ± 0.10; in control *n* = 63; 0.97 ± 0.12 and (**c**), *t* = 0.60, df = 40.00, *P* = 0.60; number of delta MAX-MIN in GF *n* = 26, 2.80 ± 0.16; in control *n* = 66; 2.90 ± 0.10). (**d**) Distribution of time intervals between two consecutive contractions (Δt) in control and GF animals. Most contractions in both GF and control polyps occur within an interval of 3 minutes (mode = 3.55 min, *n* = 787 and mode = 2.93 min, *n* = 1658, respectively). The distributions do not differ for intervals up to 15 minutes. Red-shaded area shows the time interval distribution for which GF and control significantly differ (Fisher exact test, *P* ≤ 0.001). Percent values represent the fraction of intervals taking longer than 15 min (upper line) and 30 min (lower line) in control and GF polyps (grey and red, respectively). In (**a**–**c)** boxes in beans represent the Highest Density Intervals (HDI) with 95% confidence for the mean (middle line) and the shape of the beans shows the distribution of the raw data. Data are means ± s.e.m.

To study the impact of the microbiota on spontaneous contractile behaviour, we measured the contraction frequency in germ-free (GF) animals which were obtained by treating *H. vulgaris* polyps with an antibiotic cocktail, using a regime known to completely remove associated bacteria^52^. The contraction frequency in thus obtained GF animals decreased significantly compared to controls, to an average of 4.6 ± 0.1 contractions per hour corresponding to about 60% of the control contraction frequency (Fig. 3a). To exclude that this striking difference in contraction frequency was due to disrupted epithelium contractile ability, we compared the stretching (Smax) and contractile (ΔS) capacity of the body column in control and GF polyps (Fig. 2b). This revealed no difference in stretching capacity between control and GF animals, as evidenced by similar Smax values (Fig. 3b). Likewise, there was no difference in the contractile capacity of GF and control polyps, since the changes in the polyps’ shape between a maximum stretched and the next full contraction states (ΔS, Fig. 2b) were similar (Fig. 3c). Together these findings indicate that the removal of the associated bacteria does result in a reduced number of contractions but does not impair the ability of epithelio-muscular cells of GF animals to elongate and contract.

We next analysed the distribution of time intervals between two consecutive contractions in GF animals to test whether the observed reduction was due to extended but still regular intervals between contractions. We found that the majority of the contractions of both GF and control polyps took place in a similar time frame with most contractions separated by intervals of around 3 min (Fig. 3d). Moreover, the distributions did not differ for intervals lasting up to 15 min, with 75% of all intervals being shorter than 15 min in both control and GF animals. However, a significantly larger fraction of intervals in GF polyps lasted longer than 15 min (red-shaded area in Fig. 3d), with 4% of all intervals spanning longer than 30 min. Together, these observations indicate that although most consecutive contractions in GF animals occur within normal 3 min intervals, these are intermitted by intervals lasting longer than 15 min. Thus, in the absence of bacteria the contractions seem to occur in a more irregular pattern. This suggests that symbiotic bacteria are potentially involved in supporting regular pacemaker activity and/or its transmission to the effector epithelio-muscular cells.

### Reconstitution of microbiota improves contraction frequency

**Figure 4:**
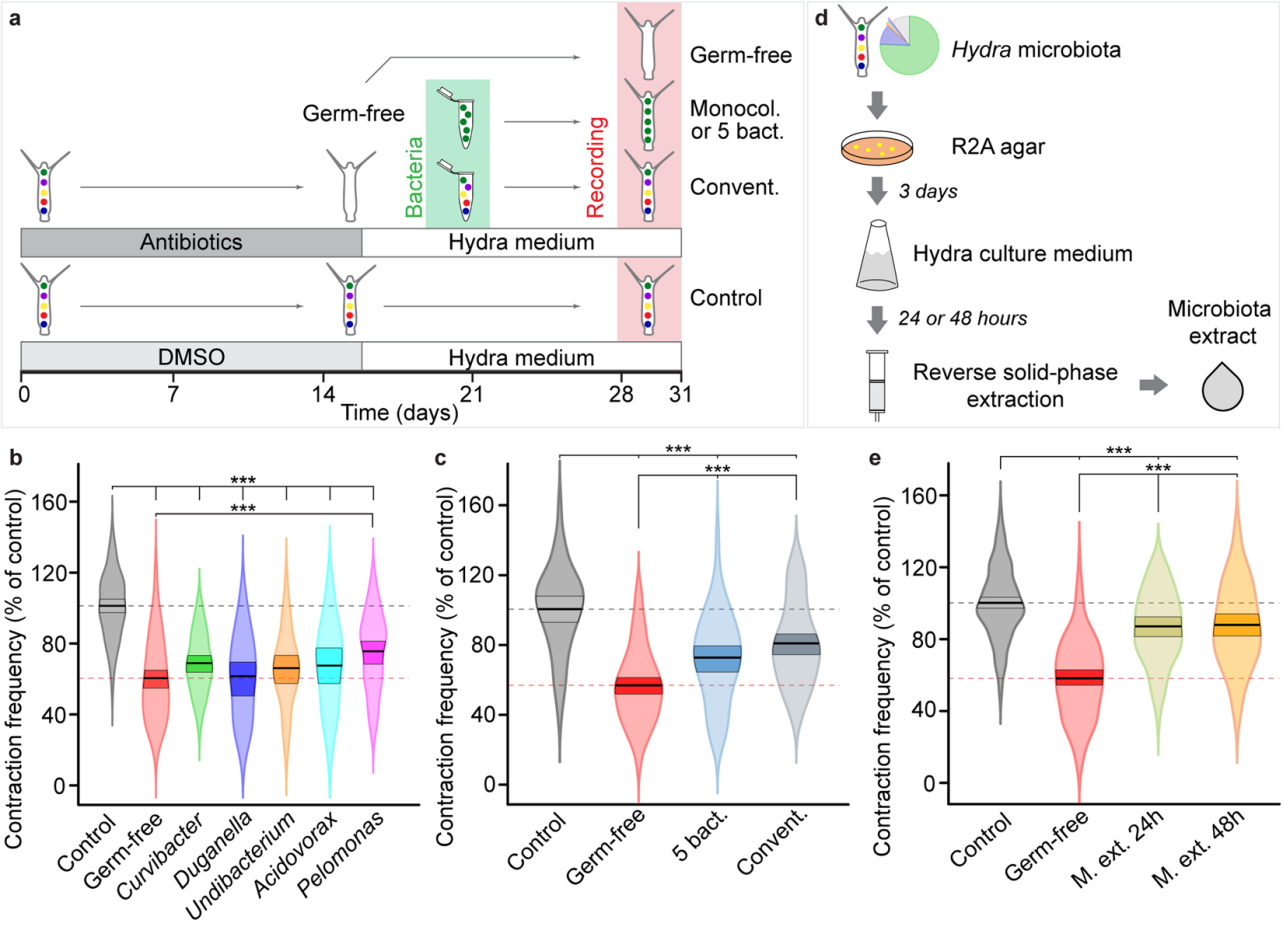
Bacterial microbiota composition affects *Hydra* spontaneous contraction behaviour. (**a**) Experimental design: GF animals were generated by antibiotic treatment and then either monocolonized with single bacterial isolates (Monocol.), with a mixture of the five main bacteria in equal proportions (5 bact.), or with natural hydra microbiota (Convent.). (**b**) Contraction frequencies of control (replicates (rep) = 8, *n* = 84), GF (rep = 8, *n* = 79) and monocolonized polyps. There is no difference in contraction frequency between GF and monocolonized animals (LME, GF compared to *Curvibacter P* = 0.09, rep = 4, *n* = 47; *Duganella P* = 0.94, rep = 3, *n* = 29; *Acid*o*vorax P* = 0.22, rep = 3, *n* = 25; *Undibacterium P* = 0.37, rep = 3, *n* = 31), except for GF and *Pelomonas* (*P* = 0.001, rep = 4, *n* = 47). All frequencies are significantly lower than those of controls (GF 58.3 ± 3.3%, *Curvibacter* 66.5 ± 3.9%, *Duganella* 58.9 ± 4.7%, *Undibacterium* 63.2 ± 4.6%, *Acidovorax* 66.1 ± 4.9% and Pelomonas 72.5 ± 3.9%; *x* ^2^ = 199, df = 6, ***P ≤ 0.001). (**c**) Contraction frequency of polyps recolonized with the five main bacteria (5 bact.) and conventionalized polyps (Convent.) is significantly higher than that of GF animals (bact. 72.8 ± 4.9%, rep = 5, *n* = 48; convent. 80.9 ± 4.6%, rep = 6, *n* = 62; GF 57.0 ± 4.4%, rep = 6, *n* = 76; anova F_(3,228)_ = 33.8, ***P ≤ 0.001). Control: rep = 6, *n* = 46. (**d**) Workflow used to obtain the microbiota supernatant extracts (M.ext.). Tissue homogenates from control polyps were plated on R2A agar and grown for three days. The resulting colonies were rinsed from the plate into sterile hydra culture medium and kept in agitation for 24 or 48 hours. The filtered supernatant was purified and concentrated using reverse solid phase extraction. (**e**) Contraction frequency of GF polyps incubated in both microbiota extracts is higher than that of GF animals incubated in hydra medium alone (M.ext.24 h 86.6 ± 3.4%, rep = 5, *n* = 49; M.ext.48 h 87.4 ± 3.4%, rep = 6, *n* = 54; GF 58.6 ± 2.6%, rep = 12, *n* = 110; anova *F*_(3,351)_ = 73.56, ***P ≤ 0.001). Control: rep = 12, *n* = 153.

To assess causality between the presence of microbiota and contraction frequency, GF polyps were recolonized with hydra commensal bacteria, and their contraction frequency was measured. The experimental approach is summarized in Fig. 4a. Since hydra’s symbiotic microbiota is relatively simple in comparison to that of vertebrates (Fig. 1d)^53^, it allows analysing the role of each member individually. First we tested the role of the main bacterial symbionts separately, by mono-colonizing GF polyps with isolates of the five strains that make up the majority of the microbiome (Fig. 1d): strains of *Curvibacter*, *Duganella*, *Undibacterium*, *Acidovorax* and *Pelomonas* genera^51^ (see *Methods*). Colonizing GF animals with single bacteria strains did not restore the contraction frequencies to control levels (Fig. 4b). In fact, frequencies of most monocolonized animals did not differ from GF values (Fig. 4b). Only recolonization with *Pelomonas* increased the contraction frequency in comparison to GF (Fig. 4b). These marginal effects of monocolonizations on contraction frequency contrasts with the striking behavioural difference between control and GF animals, and suggests that the presence of a complex bacterial ensemble may be necessary to restore the normal contractile behaviour.

We next investigated the role of the microbiota composition in modulating the contraction frequency. Two different microbial assemblages were assessed. First, GF animals were recolonized with the five main hydra bacteria strains combined in equal proportions. This composition does not mirror the *in vivo* situation, since the most abundant member (*Curvibacter*) is under-represented, while the other four (*Duganella*, *Pelomonas*, *Undibacterium*, *Acidovorax*; Fig. 1d) are over-represented. However, the rare bacterial strains (“*Others*” on Fig. 1d) are missing in this assemblage, allowing us to assess the relative contribution of rare and dominant bacteria. Animals recolonized with this 5-strain mixture showed significantly higher contraction frequency than GF polyps, although it was not completely restored to control levels (Fig. 4c).

The second assemblage used reflects more accurately the natural microbiota community in terms of strain composition and abundance (Fig. 1d) since it was directly prepared from tissue homogenates of control polyps (Fig. 4a). GF polyps recolonized with this microbiota (referred to as conventionalized) showed significantly higher contraction frequency compared to GF polyps (Fig. 4c). These animals also had a tendency to display a higher frequency than polyps recolonized with the five main bacteria (*P* = 0.067). Taken together, these results may indicate that not just the presence, but the relative abundances of the main colonizers play an important role in modulating the frequency of spontaneous contractions. Moreover, the presence of low-abundance bacterial strains may be essential for normal contractile activity. Finally, potential interactions among the members of the community may contribute to normal contraction dynamics.

### Microbiota supernatant extract improves contraction frequency in GF animals

To gain first insights into the mechanisms by which bacteria affect hydra spontaneous contractions, we prepared a microbiota supernatant extract (M.ext.), which is essentially a concentrated purified culture medium conditioned by bacteria for 24 and 48 hours (Fig. 4d). GF polyps incubated in these extracts displayed a significantly higher contraction frequency than GF animals incubated only in hydra medium, and these frequencies were only about 12% lower than the control contraction frequency (Fig. 4e). There was no difference between the contraction frequencies of polyps incubated in the 24 h and the 48 h extracts (*P* = 0.80). Since the extracts were prepared using bacteria cultures from tissue homogenates of control polyps, we assumed to have retained the natural microbiota composition and abundance, as well as the potential bacterial interactions normally occurring among undisturbed microbiota. Before the extraction, the bacterial cells were removed from the supernatant by centrifugation and filtration, therefore the supernatant was assumed to contain only molecules secreted by bacteria. Moreover, as methanol was the solvent employed for the extraction followed by a heating step at 40 °C to remove the solvent (see *Methods*), it is likely that the bacteria-secreted products present in the final extracts are polar and thermoresistant molecules. Taken together, our findings suggest that some water-soluble and small product(s) secreted by hydra-associated bacteria are involved in modulating the spontaneous contraction frequency. This is in accordance with previous views^54,55^ that small molecules such as aminoacids may influence the pacemaker activity in hydra.

## Discussion

Spontaneous and rhythmic contractile activity is observed in bilaterian animals, as well as in early-emerging metazoans. Here we analysed the interaction between the microbiota and spontaneous contractile behaviour in the cnidarian *Hydra*. We found that the bacterial microbiome directly affects the spontaneous body contraction pattern of the polyps, and that products secreted by the bacteria are likely responsible for this effect. Since the absence of microbiota affects the contraction frequency and contraction regularity but not the amplitude of the contractions (Fig. 3), we conclude that bacteria influence the pacemaker activity that is responsible for timing of the contractions. Early behavioural and electrophysiological studies on hydra showed that pacemaker activity is located in the hypostome and foot region, and suggested that the pacemakers were neurons^45–48^. Recently this idea was confirmed by functional alteration of neurons in the foot^44^ as well as by calcium imaging of neuronal activity^56^. Using genetically engineered hydra, the later study identified separate non-overlapping neuronal networks, which are associated with specific behaviours^56^. The molecular signature of the pacemaker neurons, including the receptors that would be responsive to the microbiota-derived signals remain to be characterized.

Our observations provide important insights into the mechanisms of host-microbiome interactions. First, since the phylum Cnidaria diverged before the radiation of Bilateria, the ability of the microbiota to modulate spontaneous contractile activity appears to be an evolutionary ancient feature. Second, most monocolonization experiments did not improve the contraction frequency, in contrast to conventionalized animals. This suggests that the bacterial community and interactions among its members, rather than single bacterial species, are essential for the proper function of the *Hydra* metaorganism, including normal spontaneous contraction dynamics. It has been demonstrated that such interactions among hydra’s main bacterial colonizers are critical for protecting the polyps against fungal infections^51^. The fact that the least abundant of the main members of hydra´s microbiota, *Pelomonas* sp., shows a positive effect on the contraction frequency similar to conventionalized animals, points to the important role of less abundant members of the community supporting these interactions. We note however, that the effects of monoassociations do not reflect the natural situation encountered by the host or the bacteria, and that hydra bacteria behave differently when growing *in vitro* and *in vivo* ^57^. Therefore, the effect of *Pelomonas* has to be further investigated before more conclusions can be drawn. Third, a cell-free supernatant extract from the natural microbiota culture resulted in the best improvement of the contraction frequency. This strongly suggests that a water-soluble, secreted bacterial product is involved in modulating hydra body contractions, which agrees with previous pharmacological interference studies on hydra pacemaker activity^54,55^. Moreover, it indicates that this putative secreted product is produced in absence of the host. Finally, our work highlights the importance of symbiotic microbiota in maintaining host homeostasis and supports the idea that the interaction between microbiota and neurons is truly bi-directional. A previous study has shown that hydra neurons secrete peptides that can shape the microbiota composition^58^. Here we show that hydra microbiota in turn modulates host spontaneous contraction dynamics. In agreement with this, studies in mice have demonstrated excitatory effects of the gut microbiota on the enteric nervous system^59,60^, while in zebrafish it has been shown that the enteric nervous system, acting through its control over gut motility, may influence the composition of the gut microbiota^61^.

Although there is a strong correlation between the motor activity of the gastrointestinal tract and the microbial assemblage in the gut of humans and vertebrate models, it is still unclear whether dysmotility is cause or consequence of an altered microbiota composition^62–64^. Our results provide unambiguous evidence for the casual role of bacteria in modulating spontaneous contractile behaviour, which is the basis for gut motility and the outcome of an ancient interaction between bacteria and early emerging animals. This, together with recent advances in molecular and imaging methods^37,56^, makes a strong case to consider the *Hydra* metaorganism as a strategic experimental system suitable to study basic biological principles with high translational relevance for a better understanding of gastrointestinal disorders.

## Methods

### Animals

*Hydra vulgaris* strain AEP was used in all experiments presented here. The animals were maintained according to standard procedures^65^ and fed three times per week on newly hatched *Artemia salina* nauplii. Only polyps without buds or gonads were used in the experiments. The animals were fed for the last time one day before starting the antibiotic treatment (see below). All animals used in the experiments were clones coming from the same culture dish.

### Generation of germ-free and recolonized *H. vulgaris* AEP

Germ-free (GF) polyps were obtained by treating control animals for two weeks with an antibiotic cocktail containing rifampicin, ampicillin, streptomycin and neomycin in final concentrations of 50 µg/ml each and spectinomycin at 60 µg/ml, as previously described^52^. Since rifampicin stock is dissolved in DMSO, control polyps were incubated in the corresponding final DMSO concentration (0.1%) for the same period of time. Antibiotic solution and control polyp medium was replaced every 48 hours. After 17 days in the antibiotic cocktail, the animals were transferred to sterile hydra medium that was further replaced every 48 hours until the behavioural tests were performed (days 28–31). The GF status of the polyps was tested twice per week, starting at day 19–20, by plating an antibiotic-treated macerated polyp from every replicate produced on R2A agar, which supports growth of hydra microbiota^52^. Absence of colonies following three days of incubation at 18 °C showed the GF status. GF polyp macerates were further analysed by 16S rDNA amplification, using universal 16S rRNA primers Eub-27F and Eub-1492R^66^. Absence of amplification product confirmed the GF status.

GF animals were monocolonized by incubating the polyps with a pure culture of each of the main colonizers *Curvibacter* AEP 1.3*, Duganella* C 1.2*, Undibacterium* C 1.1*, Acidovorax* AEP 1.4 and *Pelomonas* AEP 2.2. These bacteria were cultured from existing isolate stocks^51^ in R2A medium at 18 °C for three days. Approximately 5 × 10^5^ cells (based on OD_600_ quantification as previously described^51^) of each bacterial strain were added separately to 50 ml sterile hydra medium containing 20–30 GF polyps on day 20. Likewise, GF polyps were recolonized with a mixture of the five main colonizers in equal proportions (5 × 10^5^ cells /strain). After three days of incubation, the polyps were washed with and transferred to sterile hydra medium. Conventionalized animals were obtained by incubating GF polyps with tissue homogenates of control animals (one control polyp per one GF polyp) in 50 ml sterile hydra medium. The conventionalized polyps were washed and transferred to sterile hydra medium 24 hr after colonization. This medium was replaced every 48 hr until the behavioural tests were performed (days 29–31). Recolonizations were verified by plating tissue homogenates on R2A agar and by 16S rDNA amplification and sequencing (Suppl. Fig. 1) as described above.

### Production of microbiota supernatant extract

Tissue homogenates from three control polyps, previously rinsed with sterile hydra medium, were plated on R2A agar and grown at 18 °C for three days (Fig. 4d). The resulting colonies were rinsed from the agar into 500 ml sterile hydra medium and kept agitated at 18 °C for either 24 hours or 48 hours. The suspensions were centrifuged (1400 g, 10 min) and the supernatant collected, filtered (0.2 µm pores) and stored at −80 °C. The supernatants were loaded onto C18 solid phase extraction (SPE) columns (Supelco Superclean^TM^ LC-18, 60 ml, 10 g), previously equilibrated with 100% methanol followed by 10% (vol/vol) methanol, at a flow rate of 3 ml/min. Directly after loading, the columns were eluted with 100% methanol and if necessary, the elution products were stored at −80 °C prior evaporation. The eluates were dried using a rotary evaporator and a vacuum pump (40 °C, 50–100 mbar) and resuspended in 5 ml of hydra culture medium. This produced 100x-concentrated extracts that were subsequently filter-sterilized and stored at −80 °C. GF polyps were incubated in 20x diluted microbiota extracts overnight (~16 hours) before behavioural tests were performed.

### Behavioural tests

Individual polyps were placed in 200–500 µl hydra medium^65^ on microscope concave glass slides and recorded using C-mount 5-megapixel digital cameras (Breukhoven Microscopes Sytems) fitted onto M3C Wild Heerbrugg binocular microscopes. The animals were light-adapted overnight before recording and recorded in an insulated climate chamber at 18 °C, to avoid any external stimuli that could induce other than spontaneous contractions. A time series was recorded for 90 min, taking one frame every 3 seconds, with dim non-localized white light. After trimming the first 30-min acclimation period, the remaining 60-minute time series were used to quantify the contractions. Using ImageJ^67^ to visualize the time lapses, contractions were manually identified and registered with the number of the frame where they occurred. The contraction frequency expressed as number of contractions per hour was converted into percentages of the mean values of control polyps in each independent experiment (replicate). The sample size (n) reported is the total amount of animals used in each treatment (e.g. control, GF). Each animal employed was assigned to only one treatment and was recorded only once. Contraction counting was blinded to treatment (i.e. GF, recolonized and supernatant).

The time interval between two consecutive contractions Δt (Fig. 2b), was calculated in minutes. Variation in the polyps’ shape was used to quantify the stretching (Smax) and contractile (ΔS) capacity of the animals. Smax was defined as the average of the maximum elongations achieved by the polyps between two contractions (MAX, Fig. 2b), and ΔS as the average difference in the polyp’s shape between a maximum elongated state and the next contraction (MAX-MIN, Fig. 2b).

We used the radius of gyration (RoG) to assess the shape of the polyps and thus to calculate Smax and ΔS in control and GF animals. The RoG describes the shape of an object by assessing how compact it is, therefore, the more stretched an object is, the larger its RoG will be. For each image in a given time series, after converting it to black and white, the edges (i.e. the silhouette) of the polyp and its geometric center (or center of mass, gd(x,y)) were determined. An image of a polyp has as many edges as black pixels adjacent to white pixels. Using these two variables, the RoG corresponding to a given image was calculated as the average distance from the edges of the polyp to the geometric centre. See supplementary video 2. To account for size difference among the polyps, we used z-scores to standardize the RoG values corresponding to maximum elongations and full contractions (MAX and MIN respectively, Fig. 2b). The z-scores were calculated by subtracting the average and dividing by the standard deviation of all the RoG values in a given time lapse (i.e. for a given polyp). A z-score may be positive or negative, depending on whether the value is greater or smaller than the mean. A z-score equal to zero means that the value has the same value as the mean. The magnitude of the z-score also tells how many standard deviations the score is above or below the mean. The images were processed in ImageJ and the RoG values calculated using custom-made functions in R^68^.

### Statistical analysis

Differences in contraction frequency among the treatments (i.e. control, GF, recolonizations, GF incubated in the microbiota extract) were analysed using ANOVA, with treatment and replicate as explanatory variables and contraction frequency (expressed as percentage of the control) as response variable. If replicate had no effect, this variable was removed from the analysis. In cases where the variance in contraction frequency due to the replicate was greater than zero, linear mixed effects models (LME) were employed using replicate as random effect. As the replicates in each experiment were spread over more than six months, LME allows accounting for batch variability before calculating the variation in contraction frequency due to the treatment (i.e. the fixed effect). Differences in stretching (Smax) and contractile (ΔS) capacities were tested using unpaired *t*-test. Contraction interval distributions (Δt) were analysed using χ^2^ test.

All analyses were done in R^68^. LME analysis was performed using the lme4 package^69^. The p-values reported for LME are taken from ANOVA type II tests on the final models. For post-hoc analysis we used the multicomp package^70^, with false discovery rate (fdr) for p-values adjustments. Normality in the distribution of the variables was tested graphically by means of quantile-quantile (Q-Q) plots.

## Electronic supplementary material

Supplementary Video 1

Supplementary Video 2 

Supplementary Information
