# Supplementary material for: Spontaneous body contractions are modulated by the microbiome of Hydra
Source: Sci Rep. 2017 Nov 21;7:15937. doi: 10.1038/s41598-017-16191-x (PMC5698334; doi:10.1038/s41598-017-16191-x)
Supplement: Supplementary file 3 — Supplementary Information [file 41598_2017_16191_MOESM3_ESM.pdf]

Supplementary Information

Spontaneous body contractions are modulated by the microbiome of *Hydra*

Authors: Andrea P. Murillo-Rincon[1], Alexander Klimovich[1], Eileen Pemöller[1], Jan Taubenheim[1], Benedikt Mortzfeld[1], René Augustin[1], Thomas C. G. Bosch[1*]

Affiliations:

[1]Zoological Institute and Interdisciplinary Research Centre Kiel Life Science, University of Kiel, 24098 Kiel, Germany.

*Correspondence to: Thomas C. G. Bosch, Zoological Institute & Interdisciplinary Research Centre Kiel Life Science, University of Kiel, Olshausenstrasse 40, D-24098 Kiel (Germany). E-mail: tbosch@zoologie.uni-kiel.de

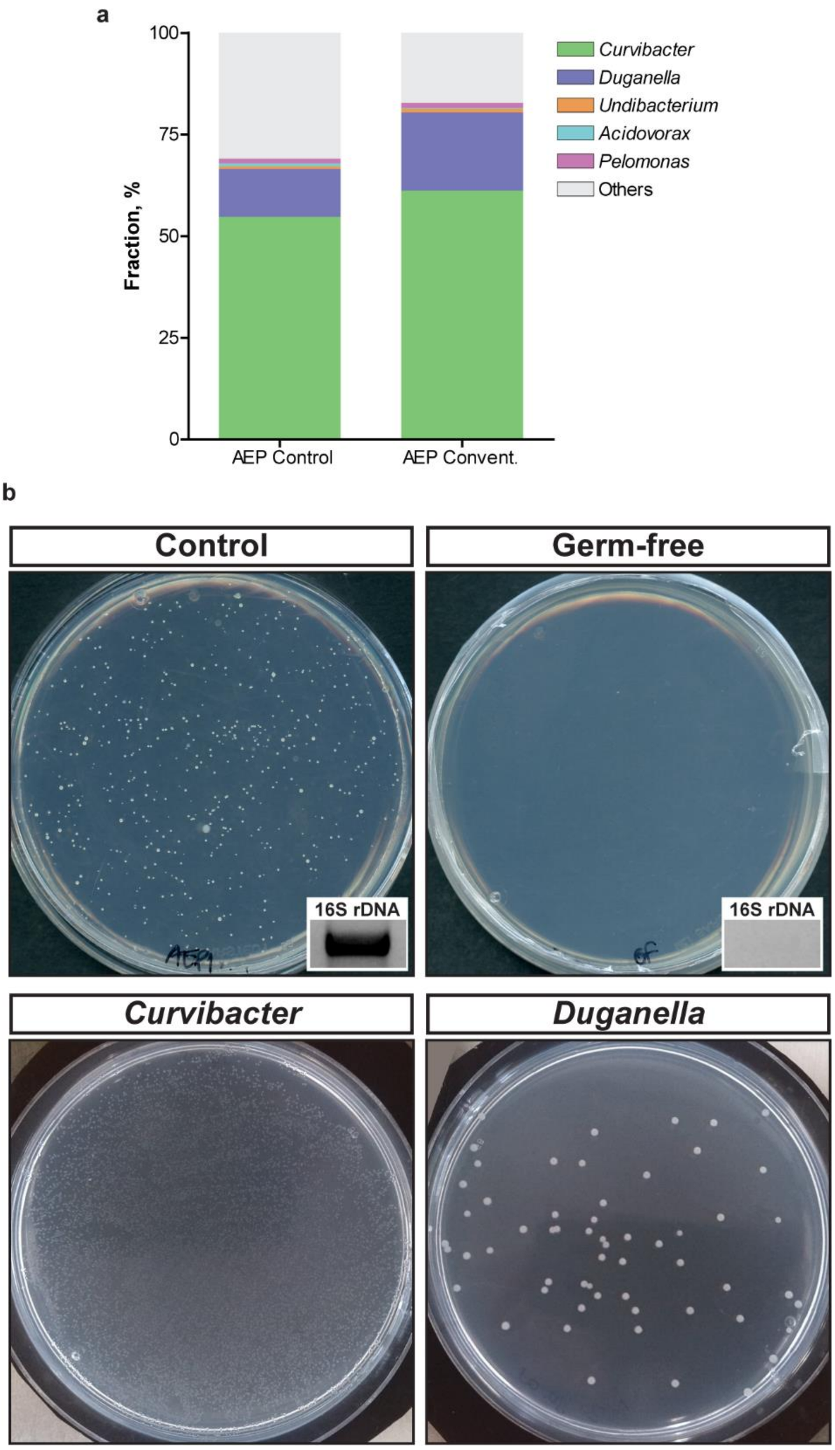

Supplementary figure1. (**a**) Recolonization of germ-free polyps results in the establishment of a bacterial community similar to that of the donor control polyp. The entire *H. vulgaris* AEP microbiota was transferred from control polyps to germ-free animals. The microbial community from the donor polyp (AEP Control, n=1) resembles previously reported natural microbiota in terms of species composition and relative abundance [1,2]. In recolonized polyps (AEP Convent., n=6), microbiota composition is remarkably similar to that of the donor polyp. Stacked bar charts represent relative abundances of the five main bacterial colonizers: *Curvibacter, Duganella, Unibacterium, Acidovorax* and *Pelomonas*. The variable regions 1 and

2 (V1V2) of the bacterial 16S rRNA genes were amplified and paired-end sequenced (2x300bp) using Illumina MiSeq platform. The analysis was conducted using Qiime 1.9 package [3]. The sequences were assembled using SeqPrep and clustered into OTUs according to 97% identity using the pick_open_reference_otus.py protocol. Chimeric sequences were identified using ChimeraSlayer [4], manually verified and removed from the dataset. OTUs were classified using the greengenes reference and OTUs with < 50 reads were removed from the dataset to avoid false positives [5]. For relative abundance, the all samples were normalized to the lowest number of reads in the dataset (22000). (**b**) Representative plates demonstrating the routine check of germ-free status and recolonization success. In each case, one macerated polyp was plated onto a R2A agar plate and incubated for three days. In control polyps (Control) colonies of diverse morphology are observed and 16SrDNA amplification provides a clear band (inset). Polyps were considered germ-free (Germ-free) if neither colonies nor band were detected. Plating of monocolonized polyps shows presence of only one colony type, as shown here for example with *Curvibacter* and *Duganella.*

Supplementary video 1

Example of *Hydra* spontaneous body contractions (65x). The spontaneous contractions are shrinkages of the body column that occur periodically in the absence of any exogenous stimuli. A full body column contraction is also called a contraction burst because it reduces the polyp to a tight ball in a series of step-wise contractions. Contraction bursts are always preceded by endogenous electrical activity generated by pacemakers in the sub-hypostome region [6,7], which are presumably modulated by another pacemaker located in the lower body column [8].

Supplementary video 2

Spontaneous contractions assessment. 60-minute time series were recorded from individual polyps. Time lapses from control and GF polyps displaying average contraction frequencies (7-8 and 4-5 contractions/hour, respectively) were randomly selected and converted into black and white images. For every image in each time series (around 1203 images/time series), the edges (i.e. the silhouette) of the polyp were detected and its geometric center calculated. The radius of gyration RoG, the average distance from the edges of the polyp to the geometric centre, was calculated for every image in a time series (i.e. 1203 RoG values/time series). When the RoG values are plotted against time, it is possible to identify full contracted and full elongated states. However to avoid false-positives, both states were manually identified. Contraction frequency, interval between two consecutive contractions, contractile and stretching capacity were evaluated as described in the Methods section.

Supplementary References

1. Franzenburg, S. *et al.* Distinct antimicrobial peptide expression determines host

species-specific bacterial associations. *Proc. Natl. Acad. Sci. U. S. A.* **110,** E3730-8 (2013).

2. Fraune, S. *et al.* Bacteria-bacteria interactions within the microbiota of the ancestral metazoan Hydra contribute to fungal resistance. *ISME J.* **9,** 1543–56 (2015).
3. Caporaso, J. G. *et al.* QIIME allows analysis of high- throughput community sequencing data Intensity normalization improves color calling in SOLiD sequencing. *Nat. Publ. Gr.* **7,** 335–336 (2010).
4. Haas, B. J. *et al.* Chimeric 16S rRNA sequence formation and detection in Sanger and 454-pyrosequenced PCR amplicons. *Genome Res.* **21,** 494–504 (2011).
5. Faith, J. J. *et al.* The long-term stability of the human gut microbiota. *Science (80-.).* **341,** 1237439 (2013).
6. Passano, L. M. & McCullough, C. B. Co-Ordinating Systems and Behaviour In Hydra: I. Pacemaker System of the Periodic Contractions. *J. Exp. Biol.* **41,** 643–664 (1964).
7. Passano, L. M. & McCullough, C. B. Pacemaker Hierarchies Controlling the Behaviour of Hydras. *Nature* **199,** 1174–1175 (1963).
8. Takaku, Y. *et al.* Innexin gap junctions in nerve cells coordinate spontaneous contractile behavior in Hydra polyps. *Sci. Rep.* **4,** 3573 (2014).
